# COVID-19 and remote consulting strategies in managing trauma and orthopaedics

**DOI:** 10.1136/postgradmedj-2020-137917

**Published:** 2020-05-13

**Authors:** Karthikeyan Iyengar, Abhishek Vaish, Eugene Toh, Raju Vaishya

**Affiliations:** Department of Orthopaedics, Southport and Ormskirk Hospital NHS Trust, Southport, UK; Department of Orthopaedics, Indraprastha Apollo Hospital, New Delhi, India; Department of Orthopaedics, Southport and Ormskirk Hospital NHS Trust, Southport, UK; Department of Orthopaedics, Indraprastha Apollo Hospital, New Delhi, India

**Keywords:** telemedicine, orthopaedic & trauma surgery, adult orthopaedics, trauma management, diagnostic radiology

As the COVID-19 pandemic took hold, the effect on healthcare systems, its resources and clinical services have been profound. With the novel coronavirus outbreak being highly contagious, there has been an ever urgent need to devise and identify new models of delivering care to avoid ‘face-to-face’ consultation between clinician and patient and thus reducing the risk of disease transmission. Managing acute trauma and orthopaedics had to be rationalised, reorganised and modified as new guidelines^1  2^ came into practice. In the UK, the National Health Service England Specialty Guides^3^ offers the primary guidance and forms the basis of all National Health Service (NHS) Trusts’ responses to this pandemic. Current and evolving telecommunication technologies play a key role in exchange of valid information for diagnosis and management of diseases and injuries. The main modalities for remote consultations include telephone consultations, virtual fracture clinics (VFC) and video consultations (VCs). Remote telephone or VCs provide a vital strategy in the delivery of trauma and orthopaedic healthcare where prevention of disease transmission, for example, current COVID-19 outbreak is of paramount importance by avoiding face-to-face consultation. However, they are appropriate in settings where a clinical interaction can occur over telecommunication channels to provide a continuity of care. Telephone consultations form the primary and readily available modality of remote access alternative to face-to-face consultations to deliver patient care.^4^ It allows most of the suitable reasons when remote consultation would be appropriate with some limitations.^5^ It can provide a means of assessing clinical condition and discussing options for managing conditions remotely, for example, assessment of pain—advice such as increasing or decreasing doses of medications or suggesting use of complimentary walking aid to improve mobility after recent surgery. It facilitates ‘risk stratifying’ a patient. For example, a patient who reports redness and pain around the postoperative wound site following recent hip surgery is at risk of surgical site infection and is actioned to be seen imminently for clinical examination. ‘Safety advice’ regarding weight-bearing status after limb surgery, for example, moving from two crutches to one following hip replacement surgery or advice regarding driving following an ankle injury can be given over telephone provided both the clinician and patient are fully aware of the boundaries of assessing patients this way. ‘Safety netting’—telephone consultations can be a means of assessing problems after interventions; for example, if a patient is being managed with a plaster of Paris cast for an ankle fracture or tendo-achilles injury, they can be risk assessed about symptoms of deep venous thrombosis (DVT), given appropriate advice regarding whom to contact according to local DVT management pathways or arrangements made for face-to-face evaluation.

VFC is essentially an extended application from telephone consultation. It is a multidisciplinary set-up and decision-making involving the clinician, physiotherapist or advance nurse practitioner to provide therapy guidance and administrative support to type letters. In the field of trauma and orthopaedics, VFCs are an alternative to conventional face-to-face fracture clinics and are increasingly being used to manage certain musculoskeletal injuries.^6^ They allow initial assessment by a senior doctor from clinical history and imaging. Following this, a remote consultation is undertaken with the patient with advice and instructions for them to ‘self-manage’ their injury. A detailed letter is sent to the general practitioner (GP) with a copy to the patient with a clear diagnosis and treatment plan in terms that can be easily understood by a layperson. On the other hand, if the clinical situation necessitates a face-to-face consultation an appointment is made in the appropriate subspecialty fracture clinic. There is an option to arrange further imaging, for example, radiographs, MRI or CT scan prior to the face-to-face appointment. This service depends on an integrated ‘electronic patient record’ and ‘picture archiving and communication imaging systems’ that can be accessed remotely to review the case. The patient would also need to be able to be easily contactable for the VFC review. Many acute musculoskeletal injuries can be managed remotely for example, posting out patient information leaflets or signposting patients to web-based resources for managing common, stable soft issue or bony injuries such as ankle sprains or metatarsal fractures of the foot. This is particularly relevant in the current COVID-19 pandemic and supported by the British Orthopaedic Association emergency specialty guidelines.^1^

NHS England and NHS improvement^7^ have increased support to encourage secondary care providers to use VCs for managing patient care in appropriate situations. VC forms a part of wider strategy for remote management of trauma and orthopaedic conditions building on telephone and VFC consultations. Various documents including user guides to support providers with rolling out of VCs have been produced by NHS England and NHS Improvement^7^ as part of national digital strategy. Complementary articles on when VC are appropriate and processes in carrying out VCs have been recently published in literature.^8  9^

With advantages of high satisfaction among staff and patients and lower transaction costs compared with traditional clinic based care, VC may have found its opportunity in reaching the next step in the ladder of remote management of patients in current pandemic.^8^

Though VC is a highly promising technology which compliments telephone remote consultations, introducing VC can be a complex organisational change.^8^ Clinician and staff reservations about technical abilities required and operational issues can be barriers. However, the advantages of VC can be significant from general applications; for example, for patients’ who do not need a physical examination and who can communicate via video to ‘triage’ and remote management of orthopaedic injuries.

Furthermore, telecommunication, webinars and virtual learning can provide education and training to clinicians about managing patients in such circumstances.^10^

In a wider context, multidisciplinary team departmental trauma meetings, normally attended by orthopaedic team members, trauma coordinators, theatre staff and anaesthetists departments, could be undertaken by remote teleconference to formulate plan of management for patients, including the ability to view radiology images and surgical planning if necessary; for example, patients with complex fractures can have planned surgery to minimise length of stay or consider day-case treatment for periarticular fractures. This allows a coordinated team approach.

Follow-up appointments can be delivered by telephone or video call in most instances especially with the current COVID-19 situation. A patient-initiated follow-up process can be organised and booked appointments should preferably be made only where it is unavoidable, for example, follow-up imaging required which may necessitate significant change in clinical management.

In managing rehabilitation services, when face-to-face meetings are likely to be limited, for example, current coronavirus outbreak, musculoskeletal triage appointments can be undertaken by the physiotherapists and occupational therapists. Patients can be given appropriate tele-advice or ‘signposted’ to alternative resources such as written or web-based information to allow rehabilitation at home, for example, rehabilitation after hip surgery or therapy goals after recent joint replacement surgery.

Medical telephone consultations have limitations; for example, inability to carry out a full clinical assessment and thereby may not be able to address a particular clinical problem or injury. Absence of visual clues and lack of physical examination are the key drawbacks. This can be overcome by enhanced documentation, shared decision-making and pragmatic management. Sending a copy of the letter to the patient and the GP, following the consultation will reinforce what was discussed in the consultation and minimise misunderstanding. Thus, though studies have reported that patients’ are equally satisfied with both forms of consultation, that is, face-to-face and telephone, they do not necessarily reduce workload for clinicians.^4^

As strategies emerge and are emerging during the COVID-19, the way we deal with managing trauma and orthopaedics is likely to change in the future with increasing use of VFC in fracture care and tele-VCs in monitoring chronic orthopaedic conditions. Evolving mobile phone technologies will take these remote consultation strategies further.
